# Plasma microRNA biomarker detection for mild cognitive impairment using differential correlation analysis

**DOI:** 10.1186/s40364-016-0076-1

**Published:** 2016-12-12

**Authors:** Mitsunori Kayano, Sayuri Higaki, Jun-ichi Satoh, Kenji Matsumoto, Etsuro Matsubara, Osamu Takikawa, Shumpei Niida

**Affiliations:** 1 Research Center for Global Agromedicine, Obihiro University of Agriculture and Veterinary Medicine, Obihiro, Hokkaido, Japan; 2 Medical Genome Center, National Center for Geriatrics and Gerontology, Obu, Aichi, Japan; 3 Department of Bioinformatics and Molecular Neuropathology, Meiji Pharmaceutical University, Kiyose, Tokyo, Japan; 4 Department of Allergy and Clinical Immunology, National Center for Child Health and Development, Setagaya, Tokyo, Japan; 5 Department of Neurology, Hirosaki University Graduate School of Medicine, Hirosaki, Aomori, Japan; 6 Department of Neurology, Oita University Faculty of Medicine, Yufu, Oita, Japan; 7 Innovation Center for Clinical Research, National Center for Geriatrics and Gerontology, Obu, Aichi, Japan

**Keywords:** Molecular network, Coexpression, Alzheimer’s disease, Dementia

## Abstract

**Background:**

Mild cognitive impairment (MCI) is an intermediate state between normal aging and dementia including Alzheimer’s disease. Early detection of dementia, and MCI, is a crucial issue in terms of secondary prevention. Blood biomarker detection is a possible way for early detection of MCI. Although disease biomarkers are detected by, in general, using single molecular analysis such as t-test, another possible approach is based on interaction between molecules.

**Results:**

Differential correlation analysis, which detects difference on correlation of two variables in case/control study, was carried out to plasma microRNA (miRNA) expression profiles of 30 age- and race-matched controls and 23 Japanese MCI patients. The 20 pairs of miRNAs, which consist of 20 miRNAs, were selected as MCI markers. Two pairs of miRNAs (hsa-miR-191 and hsa-miR-101, and hsa-miR-103 and hsa-miR-222) out of 20 attained the highest area under the curve (AUC) value of 0.962 for MCI detection. Other two miRNA pairs that include hsa-miR-191 and hsa-miR-125b also attained high AUC value of ≥ 0.95. Pathway analysis was performed to the MCI markers for further understanding of biological implications. As a result, collapsed correlation on hsa-miR-191 and emerged correlation on hsa-miR-125b might have key role in MCI and dementia progression.

**Conclusion:**

Differential correlation analysis, a bioinformatics tool to elucidate complicated and interdependent biological systems behind diseases, detects effective MCI markers that cannot be found by single molecule analysis such as t-test.

**Electronic supplementary material:**

The online version of this article (doi:10.1186/s40364-016-0076-1) contains supplementary material, which is available to authorized users.

## Background

Early detection of dementia is a crucial issue in terms of secondary prevention. Mild cognitive impairment (MCI) is an intermediate state between normal aging and dementia including Alzheimer’s disease [1–3]. On average, more than half MCI patients convert to dementia in 5 years, but some MCI patients remain stable or recover to normal over time [3–5]. This is why early detection and treatment of MCI is incredibly important.

Blood biomarkers can be useful for early detection of MCI. The present study is based on the hypothesis that neurite and synapse destruction, which are pathologic processes characteristic of early stages of AD, other neurodegenerative diseases, and MCI syndrome in general, can be detected in vitro by quantitative analysis of brain-enriched cell-free microRNA (miRNA) in the blood [6]. MiRNAs, a class of endogenous small non-coding RNAs, mediate posttranscriptional regulation of protein-coding genes by binding to the 3’ untranslated region of target mRNAs, leading to translational inhibition or mRNA destabilization or degradation [7, 8]. Overall, the whole human miRNA regulates greater than 60% of all protein-coding genes [9]. Importantly, cell-free miRNA have been shown to be stable in blood samples [10], and aberrant regulation of miRNA plays a central role in pathological events underlying cancers and neurodegenerative diseases [11–13].

A common statistical approach to detect disease biomarkers is differential expression analysis usually based on t-test between controls and patients [14]. Serum and plasma miRNA biomarkers for AD have been detected by differential expression analysis [15, 16]. Although differential expression analysis is a single molecular analysis, another possible approach is based on interaction between molecules. Such approaches, which are based on the interaction between molecules, can detect more stable and accurate biomarkers, since the interaction is array- and kit-free: a difference in mean can be easily affected by a small change in absolute expression value, but the interaction-based approach can be robust in that change. Differential correlation analysis (differential coexpression analysis, [17, 18]), an interaction-based approach, finds different types of biomarkers in terms of correlation change between controls and patients. Differential correlation has been observed in AD and cancers [19, 20].

In this paper, differential correlation analysis was carried out to plasma miRNA expression profiles of 30 age-matched controls and 23 MCI patients in Japan. Pathway analysis was performed to the detected MCI biomarkers for further understanding of biological implications of the MCI markers.

## Methods

### Participants

The use of human volunteer in this study was approved by the Ethical Review Board of Japan’s National Center for Geriatrics and Gerontology (NCGG) and the Committee of Medical Ethics of Hirosaki University School of Medicine Institutional Review Board in Japan. We used blood samples collected in NCGG Biobank and Hirosaki University School of Medicine and Hospital. Written informed consent was obtained from all participants or their family prior to the study. The characteristics of the participants are shown in Table 1: the participants were 30 age- and race-matched controls (Normal, 12 males and 18 females, mean age of 70.4) and 23 Japanese MCI patients (11 males and 12 females, mean age of 72.8). In NCGG, amnestic MCI (MCI) was diagnosed following the criteria defined by Petersen et al. [5].

**Table 1:** Summary of participants in our study. Sample size, mean age and mean score of mini mental state exam (MMSE) are shown

Class		Total	Male	Female
Age-matched controls	*#*of Participants	30	12	18
(Nornal)	Age	70.4	69.3	71.1
	MMSE	28.6	28.9	28.4
MCI patients	*#* of Participants	23	11	12
	Age	72.8	70.8	74.6
	MMSE	24.3	24.6	24.0

### Sample preparation

Total RNA was extracted from plasma using the miRNeasy Mini Kit (Qiagen) according to the manufacturer’s instructions with the following modifications. Plasma was thawed on ice and centrifuged at 3000 ×g for 5 min in a 4 °C microcentrifuge. An aliquot of 200 *μ*L of plasma per sample was transferred to a new tube and 750 *μ*L of Qiazol mixture containing 1.25 *μ*g/mL of MS2 bacteriophage RNA (Roche Applied Science) was added to the plasma. The tube was mixed and incubated for 5 min followed by the addition of 200 *μ*L chloroform. The tube was mixed, incubated for 2 min and centrifuged at 12,000 ×g for 15 min in a 4 °C microcentrifuge. The upper aqueous phase was transferred to a new microcentrifuge tube and 1.5 volume of 100% ethanol was added. The contents were mixed thoroughly and 750 *μ*L of the sample was transferred to a Qiagen RNeasy Mini spin column in a collection tube followed by centrifugation at 15,000 ×g for 30 sec at room temperature. The process was repeated until all remaining sample had been loaded. The spin column was rinsed with 700 *μ*L Qiagen RWT buffer and centrifuged at 15,000 ×g for 1 min at room temperature followed by another rinse with 500 *μ*L Qiagen RPE buffer and centrifuged at 15,000 ×g for 1 min at room temperature. A rinse step (500 *μ*L Qiagen RPE buffer) was repeated twice. The spin column was transferred to a new collection tube and centrifuged at 15,000 ×g for 2 min at room temperature. The spin column was transferred to a new microcentrifuge tube and the lid was left open for 1 min to allow the column to dry. Total RNA was eluted by adding 50 *μ*L of RNase-free water to the membrane of the spin column and incubating for 1 min before centrifugation at 15,000 ×g for 1 min at room temperature. The RNA was stored in a –80 °C freezer.

### microRNA real-time qPCR

For reverse transcription, 19.2 *μ*L of RNA eluate was used in total 80 *μ*L reactions with the miRCURY LNA™Universal RT cDNA synthesis kit (Exiqon). The cDNA products were diluted 57.25 fold (80 *μ*L cDNA reactions + 4500 *μ*L water) and assayed in 10 *μ*L PCR reactions according to the protocol for the miRCURY LNA™Universal RT microRNA PCR System; each microRNA was assayed once by qPCR on the microRNA Ready-to-Use PCR, Human panel I and panel II, V2. Negative controls excluding template from the reverse transcription reaction were assayed and profiled in the same manner of the other samples. The amplification was performed in a LightCycler®;480 Real-Time PCR System (Roche) in 384 well plates. The amplification curves were analyzed using the Roche LC software (ver. 1.5), both for determination of Cp (by the second derivative method) and for melting curve analysis.

### Data filtering

The raw data was extracted from the Light cycler 480 software. The GenEx software (Exiqon) was used for data filtering analysis. Any assay data value must be detected below Cp <37 or at least 3 Cp lower than negative control value to be included in the data analysis. Data that did not pass these criteria were omitted from any further analysis. The amplification efficiency was calculated using the LinRegPCR software. Reactions with amplification efficiency below 1.6 were also removed. All data was normalized to the average of assays detected in each sample (-dCp= average Cp [ <37] – assay Cp). We then adopted 85 miRNAs out of 745 (Table 2) detected in over 80% samples in either one of the compared two conditions followed by filtering out low expression values (<20%).

**Table 2:** 85 miRNAs in this study

hsa-let-7b	hsa-miR-142-5p	hsa-miR-186	hsa-miR-24	hsa-miR-374b
hsa-let-7d*	hsa-miR-143	hsa-miR-18a	hsa-miR-25	hsa-miR-378
hsa-let-7f	hsa-miR-144	hsa-miR-191	hsa-miR-26a	hsa-miR-423-3p
hsa-let-7g	hsa-miR-145	hsa-miR-192	hsa-miR-26b	hsa-miR-423-5p
hsa-let-7i	hsa-miR-146a	hsa-miR-197	hsa-miR-27a	hsa-miR-424
hsa-miR-101	hsa-miR-148a	hsa-miR-1979	hsa-miR-27b	hsa-miR-425
hsa-miR-103	hsa-miR-148b	hsa-miR-199a-3p	hsa-miR-29a	hsa-miR-425*
hsa-miR-106a	hsa-miR-150	hsa-miR-199a-5p	hsa-miR-29c	hsa-miR-451
hsa-miR-107	hsa-miR-151-3p	hsa-miR-19b	hsa-miR-30b	hsa-miR-484
hsa-miR-122	hsa-miR-151-5p	hsa-miR-20a	hsa-miR-30c	hsa-miR-486-5p
hsa-miR-125b	hsa-miR-152	hsa-miR-21	hsa-miR-30e	hsa-miR-505
hsa-miR-126	hsa-miR-15a	hsa-miR-22	hsa-miR-320a	hsa-miR-590-5p
hsa-miR-126*	hsa-miR-15b	hsa-miR-221	hsa-miR-320b	hsa-miR-652
hsa-miR-139-5p	hsa-miR-16	hsa-miR-222	hsa-miR-324-3p	hsa-miR-92a
hsa-miR-140-3p	hsa-miR-17	hsa-miR-223	hsa-miR-335	hsa-miR-93
hsa-miR-140-5p	hsa-miR-181a	hsa-miR-23a	hsa-miR-338-3p	hsa-miR-99a
hsa-miR-142-3p	hsa-miR-185	hsa-miR-23b	hsa-miR-342-3p	hsa-miR-99b

### Differential correlation analysis

Effective MCI markers can be found by differential correlation analysis, which investigates the difference of correlation coefficients between two classes of controls and MCI patients. In differential correlation analysis in our study, all possible miRNA pairs are ranked by the difference of two correlation coefficients

1 $$\begin{array}{@{}rcl@{}} |r_{1}-r_{2}|,  \end{array} $$

where *r*_1_,*r*_2_ are Spearman’s rank correlations of a miRNA pair for controls and MCI patients, respectively. MiRNA pairs with a high score of (1) are candidates of MCI markers.

Differential correlation analysis in our study also provides the *p*-value of a pair of miRNAs as a reference for statistical significance of the difference of correlation coefficients. For this purpose, normalized rank correlation [21, 22], *r*_*n*_, is utilized as a robust and Pearson-type correlation coefficient:

2 $$\begin{array}{@{}rcl@{}} r_{n} = \frac{\sum_{i} \Phi^{-1}\{ R_{i}/(n+1) \} \; \Phi^{-1}\{Q_{i}/(n+1)\}}{\sum_{i}\:\left[\Phi^{-1} \{i/(n+1)\}\right]^{2}},  \end{array} $$

where *Φ* is the distribution function of the standard normal distribution and *R*_*i*_ and *Q*_*i*_ are the ranks of the expression values *x*_*i*_ and *y*_*i*_ of two miRNAs, respectively. In our study, the value of normalized rank correlation *r*_*n*_ is quite similar with that of Spearman rank correlation: the mean of the difference between normalized rank correlations and Spearman’s rank correlations for all miRNA pairs was only 0.001 in our data set. Hypothesis testing to investigate the equality of two normalized rank correlation coefficients is then applied according to a likelihood ratio test in [23, 24]. The *p*-value can be calculated through the hypothesis testing. We used Spearman’s rank correlation for the difference calculation on correlation coefficients (1) and used the normalized rank correlation for *p*-value calculation.

Evaluation of the performance of a miRNA pair as MCI marker is not straight-forward. We here apply receiver-operator characteristic (ROC) analysis on logistic regression with an interaction term of two miRNAs:

3 $$\begin{array}{@{}rcl@{}} \log \frac{p}{1-p} = \beta_{0} + \beta_{1} X_{1} + \beta_{2} X_{2} + \beta_{12} X_{1} X_{2}  \end{array} $$

where *p* is the probability that a sample is in MCI class, *β*_0_,*β*_1_,*β*_2_,*β*_12_ are regression coefficients and *X*_1_,*X*_2_ are the expression value of two miRNAs, respectively. The interaction term *β*_12_ *X*_1_ *X*_2_ is essential for the evaluation of two miRNAs detected by differential correlation analysis. If the correlation coefficient between *X*_1_ and *X*_2_ is altered between controls and MCI class, then the interaction term significantly affects the discrimination of MCI from controls. The area under the curve (AUC) value (=0 to 1) is estimated through ROC analysis based on the estimated probabilities $\hat {p}_{1},....,\hat {p}_{n}$ for all samples of controls and MCI patients. If the estimated probabilities for controls and MCI patients are much different (e.g., $\hat {p}<0.5$ for controls and $\hat {p}>0.5$ for MCI patients), then AUC value will be 1 (completely separated). In order to evaluate of the performance of several pairs of miRNAs as MCI markers, logistic regression with multiple interaction terms can be available:

4 $$ \begin{aligned} \log \frac{p}{1-p} & = \beta_{0} + \beta_{1} X_{1} + \beta_{2} X_{2} + \ldots \\ & \quad + \beta_{k} X_{k} + \sum_{(i,j)\in C} \beta_{ij} X_{i}X_{j}  \end{aligned}  $$

where *C* is a set of miRNA pairs that are differentially correlated between controls and MCI patients. For example, four miRNA pairs (miRNA 1-2, 1-3, 3-4 and 4-5) with five miRNAs can be incorporated in the logistic regression model, log*p*/(1−*p*)=*β*_0_+*β*_1_ *X*_1_+*β*_2_ *X*_2_+*β*_3_ *X*_3_+*β*_4_ *X*_4_+*β*_5_ *X*_5_+*β*_12_ *X*_1_ *X*_2_+*β*_13_ *X*_1_ *X*_3_+*β*_34_ *X*_3_ *X*_4_+*β*_45_ *X*_4_ *X*_5_, where *C*={(1,2),(1,3),(3,4),(4,5)} in the interaction terms $\sum _{(i,j)\in C} \beta _{ij} X_{i}X_{j}$. ROC analysis evaluates the performance of the five miRNAs as MCI markers simultaneously.

## Results

### Differential correlation analysis

**Fig. 1:**
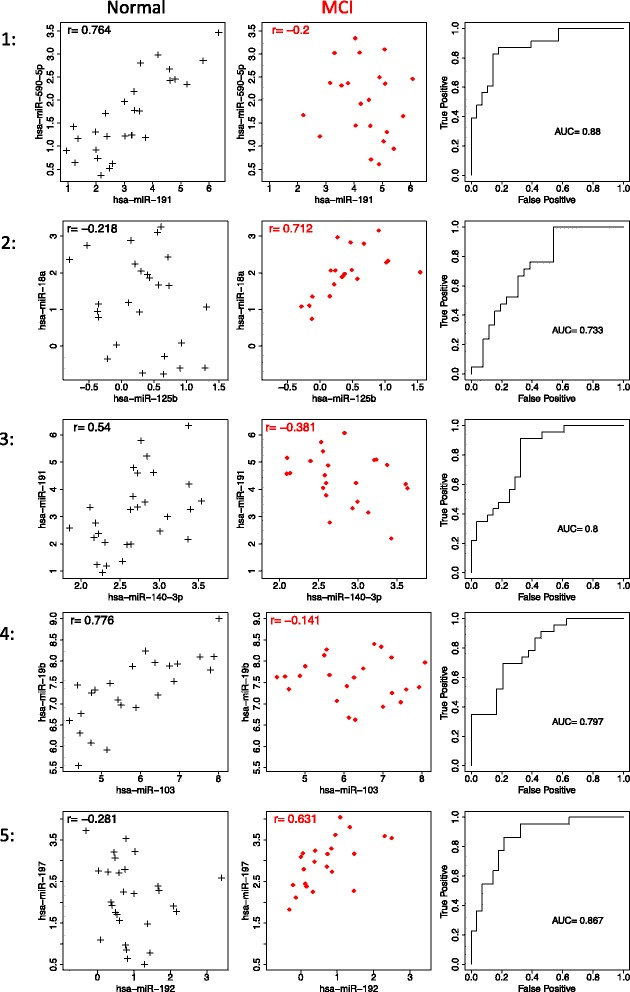
Scatterplots and ROC curves for top five miRNA pairs selected by differential correlation analysis. *Left*: Normal, *Middle*: MCI, *Right*: ROC curve

**Fig. 2:**
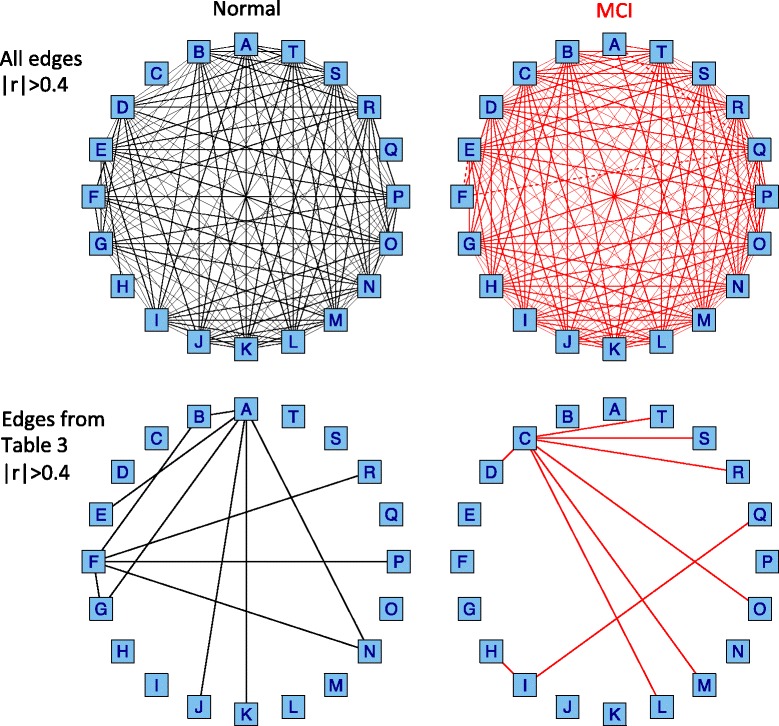
Correlation networks for the 20 miRNAs detected by differential correlation analysis. Each *box* indicates a miRNA with the alphabet in Table 5. For example, A: hsa-miR-191, B: hsa-miR-590-5p, C: hsa-miR-125b, D: hsa-miR-18a, E: hsa-miR-140-3p and F: hsa-miR-103. The 10 miRNAs (A, B, E, F, G, J, K, N, P, R) and the 11 miRNAs (C, D, H, I, L, M, N, O, Q, R, S, T) are highly correlated with each other in Normal and MCI, respectively. *Upper*: all edges with the correlation coefficient of |*r*|>0.40. *Lower*: the edges with the correlation coefficient of |*r*|>0.40 only for differentially correlated miRNA pairs in Table 3. *Solid* and *broken lines* indicate positive and negative correlations, respectively

Differential correlation analysis was applied to the data set with 85 miRNAs for age-matched samples of 30 controls and 23 MCI patients (Tables 1 and 2). The 3570 possib le pairs from the 85 miRNAs were ranked, according to the difference of correlation coefficients between controls and MCI patients. The 20 pairs of miRNAs, which had the difference of correlation coefficients of |*r*_1_−*r*_2_|>0.8, were selected as biomarkers that distinguish MCI patients from controls (Table 3). The AUC value by each of the 20 miRNA pairs was 0.800 ± 0.051 ranged between 0.718 and 0.880. Figure 1 shows scatterplots and ROC curves for each of top five miRNA pairs selected by differential correlation between normal and MCI (see also Additional file 1 for the remained miRNA pairs). Figure 2 shows correlation networks of the 20 miRNA pairs.

**Table 3:** Summary of the 20 pairs of miRNAs detected by differential correlation between Normal and MCI. The miRNA pairs are ranked by the difference of the correlation coefficients. The mean AUC value for the 20 miRNA pairs is 0.800 ± 0.051

Rank	Pair of miRNAs		|*r*_1_−*r*_2_|	log10	AUC	Correlation	Coefficients
				*p*-value		Normal (*r*_1_)	MCI (*r*_2_)
1	**hsa-miR-191**	**hsa-miR-590-5p**	0.963	-3.76	0.880	0.764	-0.200
2	**hsa-miR-125b**	**hsa-miR-18a**	0.930	-3.55	0.733	-0.218	0.712
3	**hsa-miR-140-3p**	hsa-miR-191	0.921	-2.85	0.800	0.540	-0.381
4	hsa-miR-103	hsa-miR-19b	0.917	-3.56	0.797	0.776	-0.141
5	hsa-miR-192	hsa-miR-197	0.912	-3.61	0.867	-0.281	0.631
6	**hsa-miR-191**	hsa-miR-19b	0.911	-4.10	0.854	0.826	-0.085
7	hsa-miR-152	**hsa-miR-191**	0.892	-3.42	0.863	0.772	-0.121
8	hsa-miR-103	**hsa-miR-590-5p**	0.888	-3.24	0.749	0.614	-0.275
9	**hsa-miR-191**	hsa-miR-320a	0.873	-3.38	0.872	0.691	-0.182
10	**hsa-miR-125b**	hsa-miR-20a	0.871	-3.80	0.801	-0.090	0.781
11	hsa-miR-106a	**hsa-miR-125b**	0.869	-3.94	0.785	-0.083	0.786
12	hsa-miR-101	hsa-miR-103	0.865	-3.65	0.768	0.805	-0.060
13	**hsa-miR-125b**	hsa-miR-24	0.840	-3.42	0.801	-0.073	0.768
14	hsa-miR-101	**hsa-miR-191**	0.831	-3.96	0.871	0.822	-0.009
15	hsa-miR-103	hsa-miR-222	0.828	-3.24	0.745	0.622	-0.207
16	hsa-miR-197	hsa-miR-378	0.820	-2.78	0.810	-0.234	0.586
17	hsa-miR-103	hsa-miR-223	0.815	-3.79	0.786	0.840	0.025
18	**hsa-miR-125b**	hsa-miR-223	0.815	-3.49	0.765	-0.015	0.800
19	hsa-let-7b	**hsa-miR-125b**	0.811	-3.75	0.718	-0.056	0.755
20	**hsa-miR-125b**	hsa-miR-484	0.801	-3.52	0.739	-0.078	0.723

Bold: top five miRNAs

**Fig. 3:**
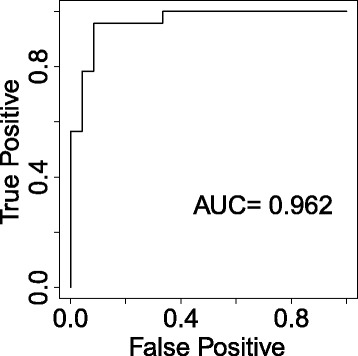
ROC curve based on the top two-pairs of miRNAs with four miRNAs (hsa-miR-191, hsa-miR-101, hsa-miR-103 and hsa-miR-222) selected by differential correlation analysis. The four miRNAs attained the highest AUC value of 0.962

AUC value for all two-pairs of the 20 miRNA pairs was also calculated by using (4). Table 4 shows summary of the top 10 two-pairs of miRNAs out of 190 possible pairs. Two miRNA pairs (hsa-miR-191 and hsa-miR-101, and hsa-miR-103 and hsa-miR-222) attained the highest AUC value of 0.962 for MCI detection (Fig. 3). Other two miRNA pairs that include hsa-miR-191 and hsa-miR-125b also attained high AUC value of ≥ 0.95 (Table 4).

**Table 4:** Summary of the top 10 two-pairs of miRNAs out of the 20 miRNA pairs detected by differential correlation analysis in Table 3. The two-pairs of miRNAs are ranked by AUC value

Rank	AUC	Original	Original	Two-pairs of miRNAs		Correlation	Coefficients
		Rank*	AUC*			Normal (*r*_1_)	MCI (*r*_2_)
1	0.962	14	0.871	hsa-miR-101	**hsa-miR-191**	0.822	-0.009
		15	0.745	hsa-miR-103	hsa-miR-222	0.622	-0.207
2	0.959	5	0.867	hsa-miR-192	hsa-miR-197	-0.281	0.631
		14	0.871	hsa-miR-101	**hsa-miR-191**	0.822	-0.009
3	0.958	6	0.854	**hsa-miR-191**	hsa-miR-19b	0.826	-0.085
		17	0.786	hsa-miR-103	hsa-miR-223	0.840	0.025
4	0.957	1	0.880	**hsa-miR-191**	**hsa-miR-590-5p**	0.764	-0.200
		17	0.786	hsa-miR-103	hsa-miR-223	0.840	0.025
5	0.957	14	0.871	hsa-miR-101	**hsa-miR-191**	0.822	-0.009
		16	0.810	hsa-miR-197	hsa-miR-378	-0.234	0.586
6	0.952	12	0.768	hsa-miR-101	hsa-miR-103	0.805	-0.060
		13	0.801	**hsa-miR-125b**	hsa-miR-24	-0.073	0.768
7	0.951	5	0.867	hsa-miR-192	hsa-miR-197	-0.281	0.631
		9	0.872	**hsa-miR-191**	hsa-miR-320a	0.691	-0.182
8	0.951	14	0.871	hsa-miR-101	**hsa-miR-191**	0.822	-0.009
		17	0.786	hsa-miR-103	hsa-miR-223	0.840	0.025
9	0.951	1	0.880	**hsa-miR-191**	**hsa-miR-590-5p**	0.764	-0.200
		15	0.745	hsa-miR-103	hsa-miR-222	0.622	-0.207
10	0.947	4	0.797	hsa-miR-103	hsa-miR-19b	0.776	-0.141
		14	0.871	hsa-miR-101	**hsa-miR-191**	0.822	-0.009

^*^: Original rank and AUC of each pair of miRNAs in Table 3. Bold: top five miRNAs

**Table 5:** The two sets of miRNAs detected by Left: differential correlation analysis and Right: t-test

Differential correlation				t-test	
20 miRNAs				22 miRNAs	
A:	hsa-miR-191	L:	hsa-miR-20a	**hsa-miR-151-3p**	hsa-miR-15b
B:	**hsa-miR-590-5p**	M:	hsa-miR-106a	hsa-miR-126*	hsa-let-7d*
C:	**hsa-miR-125b**	N:	hsa-miR-101	**hsa-miR-23a**	hsa-miR-197
D:	**hsa-miR-18a**	O:	hsa-miR-24	**hsa-miR-27b**	hsa-miR-30b
E:	**hsa-miR-140-3p**	P:	hsa-miR-222	**hsa-miR-146a**	hsa-miR-185
F:	hsa-miR-103	Q:	hsa-miR-378	hsa-miR-30c	hsa-miR-191
G:	hsa-miR-19b	R:	hsa-miR-223	hsa-miR-151-5p	hsa-miR-26b
H:	hsa-miR-192	S:	hsa-let-7b	hsa-miR-23b	hsa-miR-223
I:	hsa-miR-197	T:	hsa-miR-484	hsa-miR-92a	hsa-miR-26a
J:	hsa-miR-152			hsa-miR-24	hsa-miR-16
K:	hsa-miR-320a			hsa-miR-144	hsa-let-7f

Bold: top five miRNAs in each analysis

Underline: same miRNAs in left and right sides

The alphabets in the left side are utilized in Fig. 2

### Pathway analysis

**Fig. 4:**
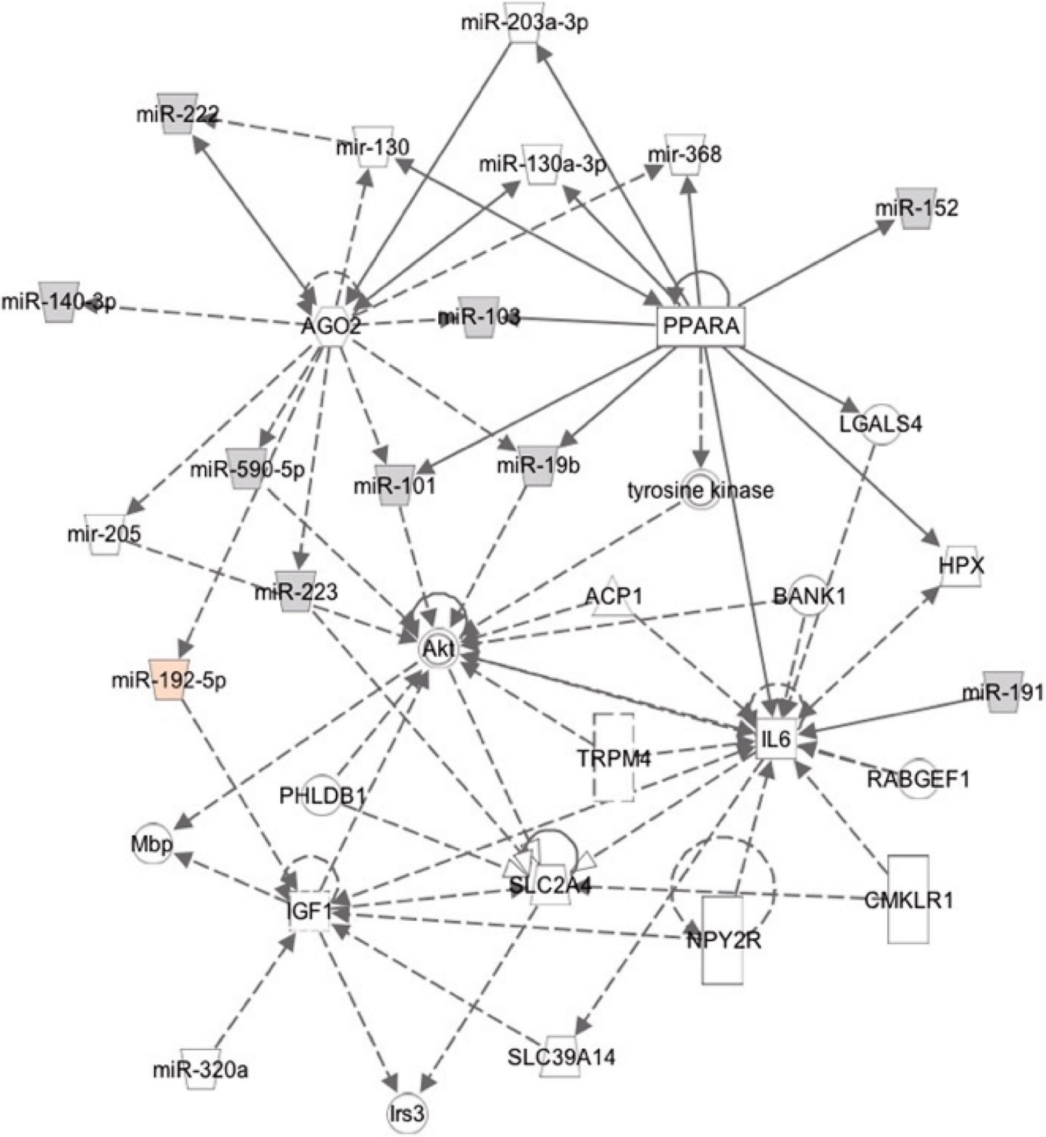
An estimated network through IPA on the 10 highly correlated miRNAs in Normal. Genes/miRNAs directly (*solid arrow*) and indirectly (*broken arrow*) interacted with them

**Fig. 5:**
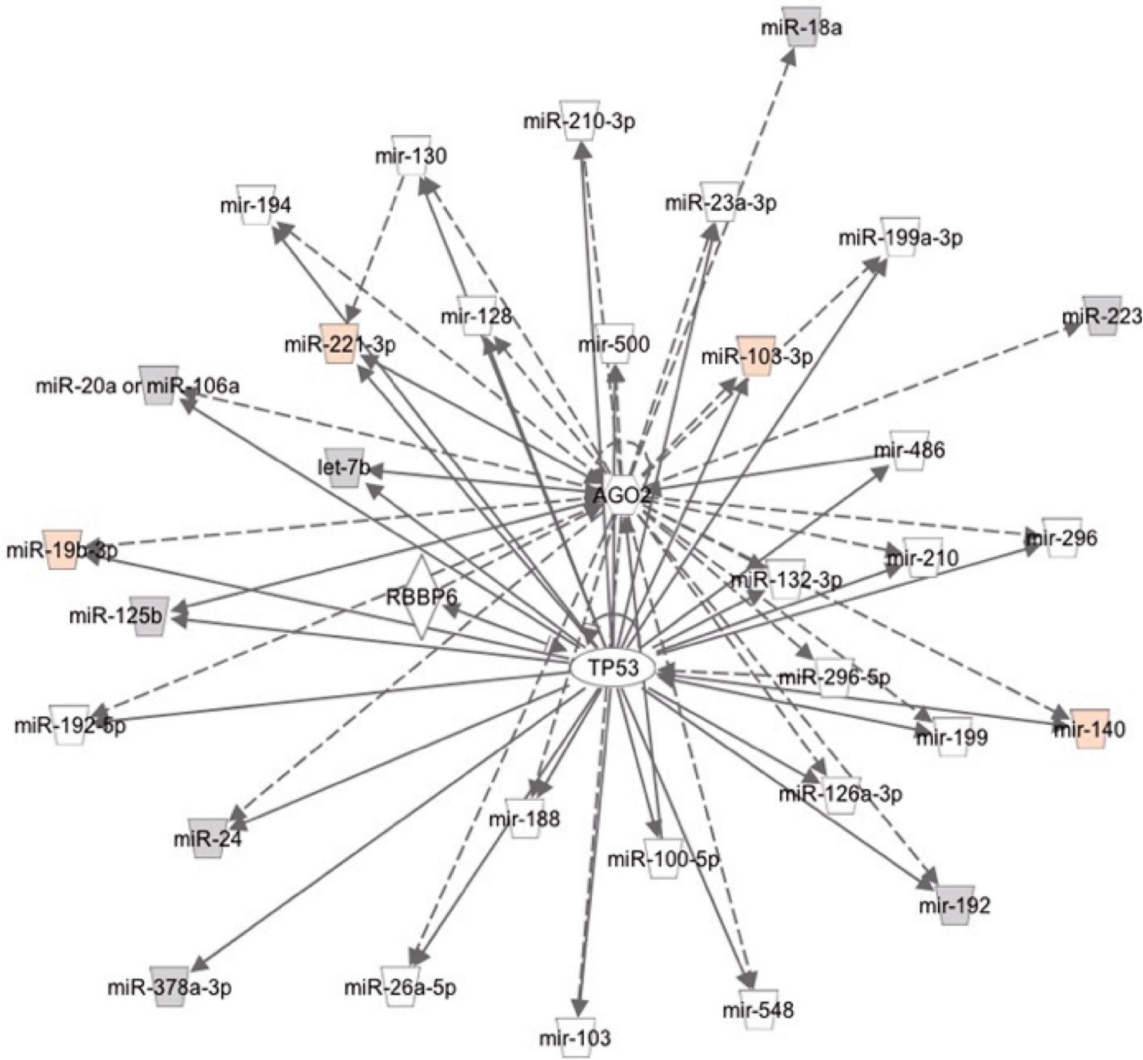
An estimated network through IPA on the 11 highly correlated miRNAsin MCI. Genes/miRNAs directly (*solid arrow*) and indirectly (*broken arrow*) interacted with them

We performed Ingenuity Pathway Analysis (IPA) about correlation networks to be lost and emerged in the MCI. Figures 4 and 5 show estimated networks through IPA on the 10 and 11 miRNAs, which are highly correlated with each other in Normal and MCI respectively. IPA revealed that the 10 highly correlated miRNAs in Normal were composed of networks surrounding Akt, IGF1, PPARA, IL6 and AGO2 genes. The IPA showed that TP53 genes directly regulated all of 11 highly correlated miRNAs in MCI. Pathways enriched for target genes of 10/11 highly correlated miRNAs in Normal/MCI are shown in Tables 6 and 7.

**Table 6:** Pathways enriched for target genes of 10 highly correlated miRNAs in Normal

# KEGG pathway	*p*-value	#genes	#miRNAs
1 Pathways in cancer (hsa05200)	2.13 ×10^−19^	101	9
2 Prostate cancer (hsa05215)	3.39 ×10^−17^	35	9
3 PI3K-Akt signaling pathway (hsa04151)	1.92 ×10^−15^	94	9
4 mTOR signaling pathway (hsa04150)	6.11 ×10^−14^	27	9
5 Insulin signaling pathway (hsa04910)	1.18 ×10^−13^	46	8
6 Endometrial cancer (hsa05213)	4.34 ×10^−13^	22	9
7 Ubiquitin mediated proteolysis (hsa04120)	6.03 ×10^−13^	46	10
8 Focal adhesion (hsa04510)	5.48 ×10^−12^	60	9
9 Non-small cell lung cancer (hsa05223)	5.16 ×10^−10^	21	8
10 Hedgehog signaling pathway (hsa04340)	6.68 ×10^−10^	20	7

**Table 7:** Pathways enriched for target genes of 11 highly correlated miRNAs in MCI

# KEGG pathway	*p*-value	#genes	#miRNAs
1 MAPK signaling pathway (hsa04010)	1.81 ×10^−13^	79	11
2 Endocytosis (hsa04144)	5.43 ×10^−12^	63	10
3 TGF-beta signaling pathway (hsa04350)	5.43 ×10^−12^	31	10
4 PI3K-Akt signaling pathway (hsa04151)	3.88 ×10^−10^	91	11
5 Pathways in cancer (hsa05200)	4.62 ×10^−10^	92	11
6 Neurotrophin signaling pathway (hsa04722)	5.66 ×10^−8^	38	11
7 Prostate cancer (hsa05215)	6.03 ×10^−8^	30	10
8 Ubiquitin mediated proteolysis (hsa04120)	1.37 ×10^−7^	41	11
9 ErbB signaling pathway (hsa04012)	5.11 ×10^−7^	29	10
10 Hepatitis B (hsa05161)	6.96 ×10^−7^	41	11

### T-test

Traditional t-test was applied to the same data set with 85 miRNAs for age-matched samples of 30 controls and 23 MCI patients (Tables 1 and 2). The detail was described in Additional file 2. The 22 miRNAs out of 85 were detected as MCI markers (Table 5 and Additional file 2). The AUC value by each of the 22 miRNAs was 0.784 ± 0.017 ranged between 0.748 and 0.828. Importantly, differential correlation analysis detected much different and more sensitive MCI markers compared to t-test (Table 5): mean AUC value = 0.800 ± 0.051 (differential correlation analysis), = 0.784 ± 0.017 (t-test). Also, the highest AUC value of any four miRNAs from the 22 miRNAs (Figure 4 in Additional file 2) was less than the highest in the two-pair approach, which investigate the performance of three to four miRNAs simultaneously, in differential correlation analysis.

## Discussion

Pathway analysis, IPA, allows us for further understanding of biological implications of the detected 20 MCI maker pairs of miRNA. Validation study and brain-based previous studies can support the results of differential correlation analysis and IPA.

IPA showed that 10 highly correlated miRNAs in Normal were composed of networks surrounding Akt, IGF1, PPARA, IL6 and AGO2 genes (Fig. 4). Akt, IGF1 and Irs3 are key molecules in insulin signaling pathway and PPARA is a regulator of lipid metabolism. Moreover, insulin, mTOR and PI3K-Akt signaling pathway were ranked among top 5 analyzed by DIANA-miRPath, which predicted miRNA targets through DIANA-microT-CDS and combined their interactions into KEGG pathway (Table 6). These pathways included target genes of 9 miRNAs except for miR-191. Previous studies consistently reported that identified biomarkers, changed genes and networks in AD patients or AD model were involved in insulin-related signaling [8, 25, 26]. Indeed, experimentally validated evidences support key role of miR-103a-3p, miR-320a and miR-590-5p in metabolic pathway [27, 28] and miR-103a-3p association with AD [29–31]. In Fig. 2, we found that miR-103a-3p and miR-191 served as hub miRNAs of 12 edges of pair correlations in Normal. miR-191 is also a widely used biomarker for diseases like cancers, type-2 diabetes and AD [32]. Considering the significant upregulation of miR-191 in MCI (t-test), these findings supposed that MCI stage lost miRNA correlations as cause and/or effect of changed expression balance among miR-191 and members in insulin related signaling. Lost of their correlation could become a discriminative marker for MCI.

There are newly emerged correlation network with a hub miRNA, miR-125b in MCI patient plasma. The IPA showed that TP53 genes directly regulated all of 11 highly correlated miRNAs in MCI (Fig. 5). TP53 has been explored originally as a tumor suppressor, but recently reported about other aspects to control diseases such as aging and metabolism [33]. There are accumulated studies that the change of TP53 protein, its modification and conformation were observed in AD patient brains [34–36] and blood [37]. Intriguingly, Le et al. demonstrated that miR-125b bound to 3’ untranslated region of TP53 mRNA and worked as a negative regulator of TP53 [38], which means a possible presence of negative feedback loop. The result of DIANA-miRPath indicated that MAPK, TGF-beta and Neurotrophin signaling pathway were characteristic in MCI, although there were overlapped pathways in Normal and MCI (Table 7). Similarly to TP53 signaling, these pathways have common biological functions such as cell survival, cell cycle and apoptosis. In this study, change of TP53 function might be detected as generated new correlations of the downstream miRNAs.

This study focuses on biomarker detection for MCI, not on mechanism that how were plasma miRNAs produced from brain. However, brain-based studies also support reliability of hsa-miR-191 and hsa-125b as MCI markers. For example, expression change of miR-191 is required for maintenance of spine restructuring in mouse hippocampus [39], and miR-125b effects on dendritic spine morphology and synaptic physiology in hippocampal neurons of mouse [40], where it has been shown that MCI and AD is a synaptic failure [41–43].

In summary, collapsed correlation on hsa-miR-191 and emerged correlation on hsa-miR-125b might have key role in MCI, and dementia progression.

## Conclusions

Differential correlation analysis, which detects difference of correlation in case/control study, was carried out to plasma miRNA expression profiles of 30 age- and race-matched controls and 23 Japanese MCI patients. The 20 miRNA pairs were selected as biomarkers for MCI. The 20 miRNAs were more sensitive and different from that by t-test.

Pathway analysis showed that, in particular, collapsed correlation on hsa-miR-191 and emerged correlation on hsa-miR-125b might have key role in MCI, and dementia progression. Differential correlation analysis detects effective MCI markers that cannot be found by single molecule analysis such as t-test. Also, differential correlation analysis could be a key bioinformatics tool to find sensitive biomarkers and to elucidate complicated biological systems behind diseases.

## Additional files

Additional file 1 Supplement A. Scatterplots and ROC curves for each of top 20 pairs of miRNAs selected by differential correlation analysis between Normal and MCI. (PDF 63.2 kb)

Additional file 2 Supplement B. Details of t-test and the results. The results include boxplots and ROC curves based on each of 22 miRNAs selected by t-test between Normal and MCI. (PDF 55.1 kb)

## Abbreviations

AD: Alzheimer’
s disease; AUC: Area under the (ROC) curve
IPA: Ingenuity pathway analysis
MCI: Mild cognitive impairment
miRNA: MicroRNA
MMSE: Mini mental state exam
NCGG: National center for geriatrics and gerontology
ROC: Receiver-operator characteristic
