# Supplementary material for: Plasma microRNA biomarker detection for mild cognitive impairment using differential correlation analysis
Source: Biomark Res. 2016 Dec 12;4:22. doi: 10.1186/s40364-016-0076-1 (PMC5151129; doi:10.1186/s40364-016-0076-1)
Supplement: Additional file 1 — Supplement A. Scatterplots and ROC curves for each of top 20 pairs of miRNAs selected by differential correlation analysis between Normal and MCI. (PDF 63.2 kb) [file 40364_2016_76_MOESM1_ESM.pdf]

Supplement A

Figures 1 to 4 show scatterplots and ROC curves for each of top 20 miRNA pairs selected by differential correlation analysis between Normal and MCI.

1:

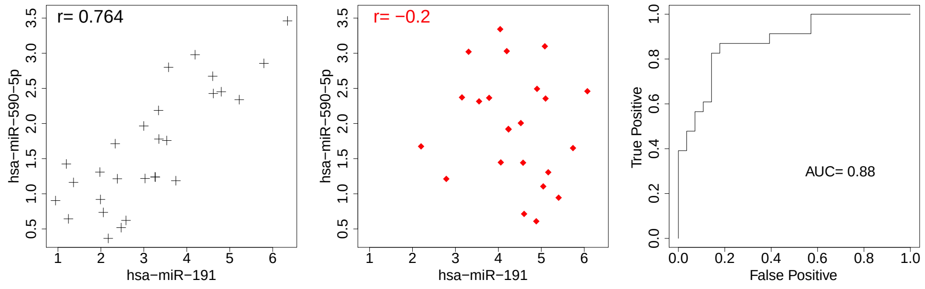

2:

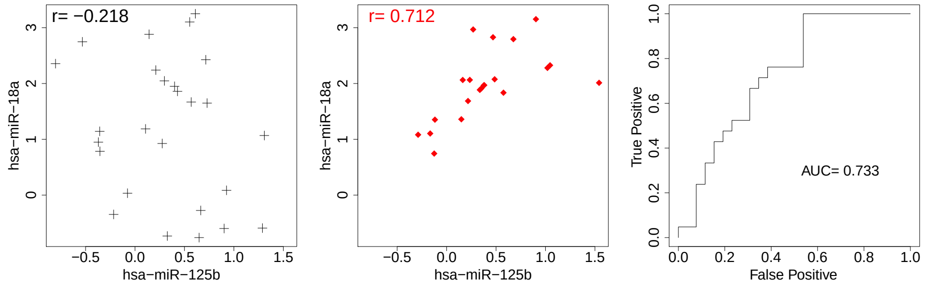

3:

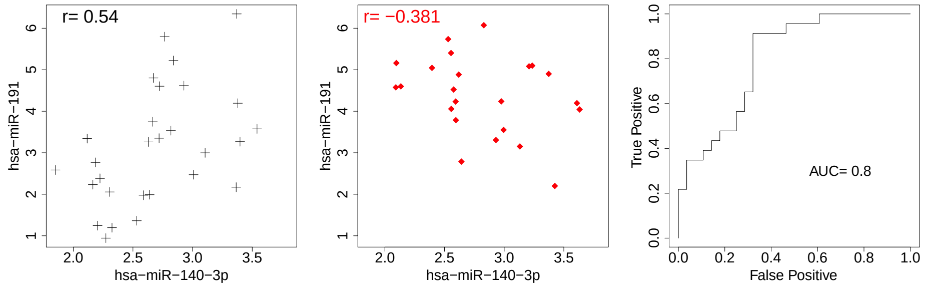

4:

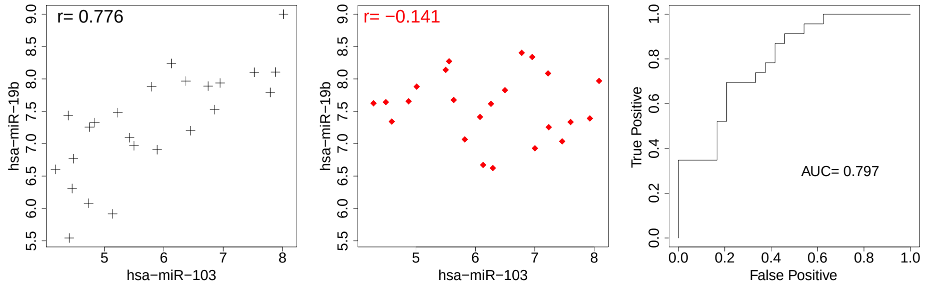

5:

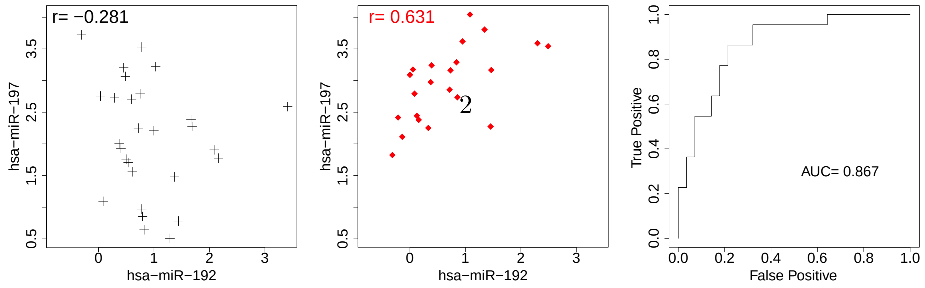

Figure 1: The scatterplots and ROC curves for the pairs of miRNAs selected by differential correlation analysis (part 1)

6:

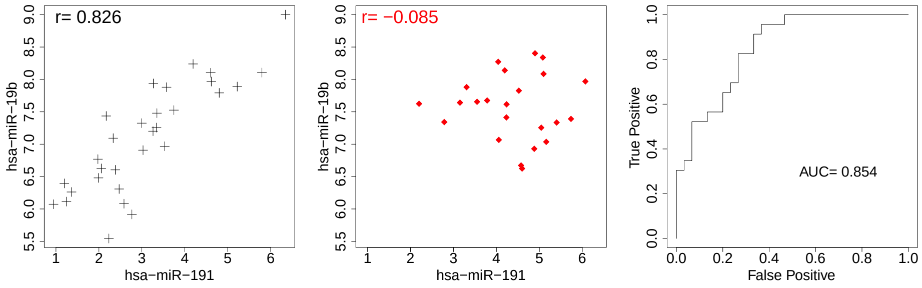

7:

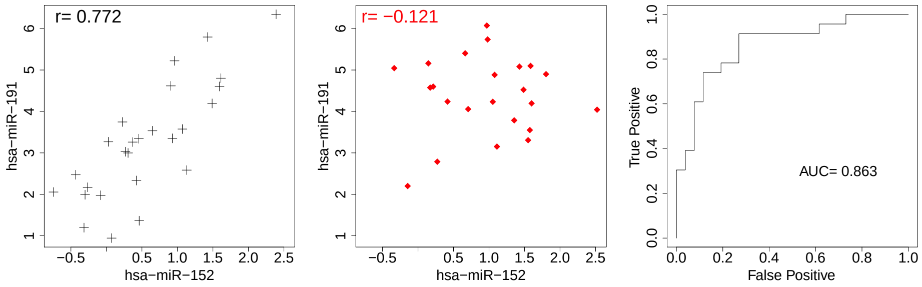

8:

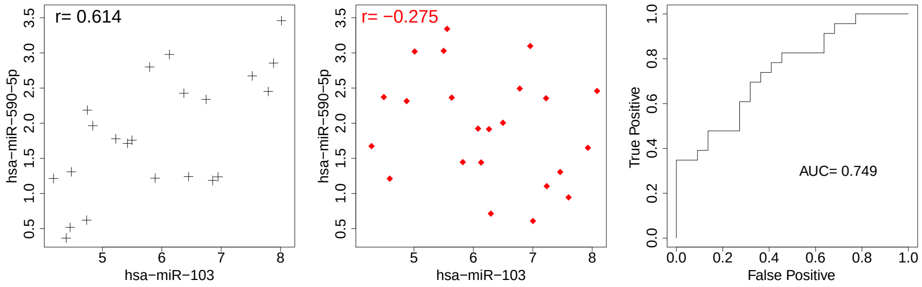

9:

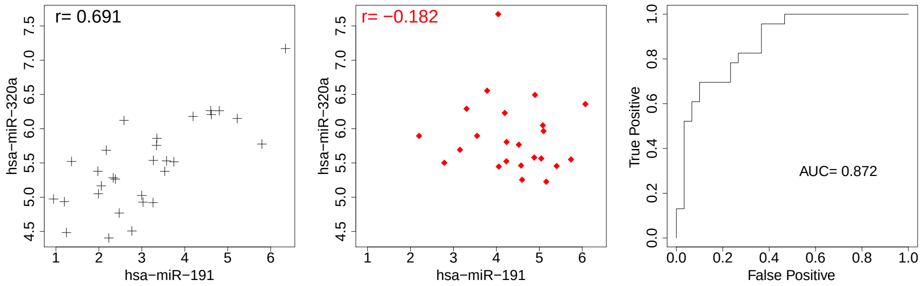

10:

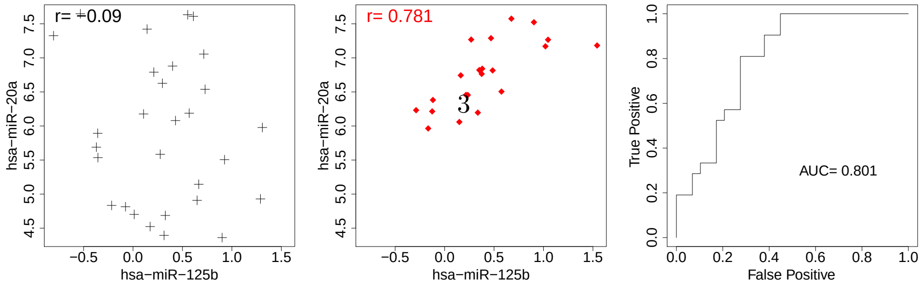

Figure 2: The scatterplots and ROC curves for the pairs of miRNAs selected by differential correlation analysis (part 2)

11:

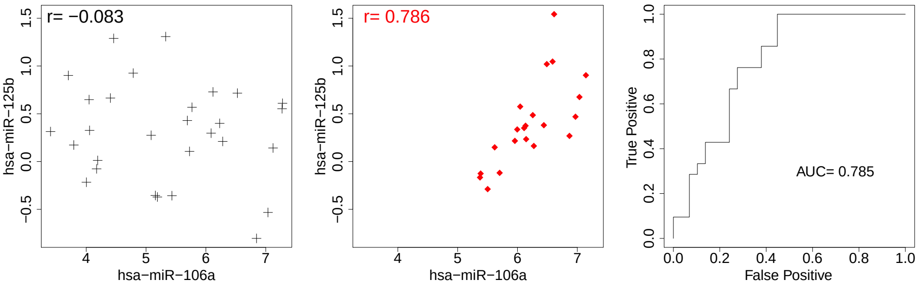

12:

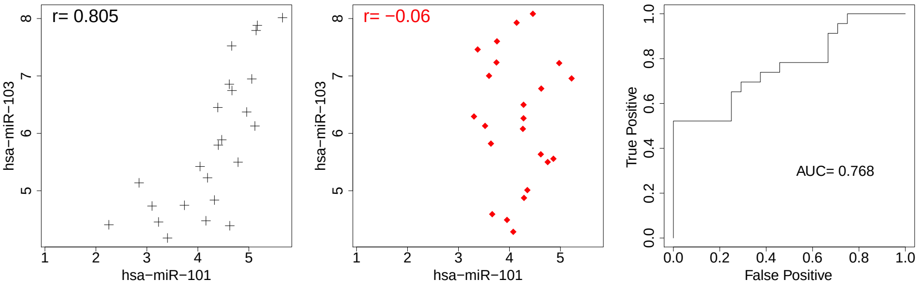

13:

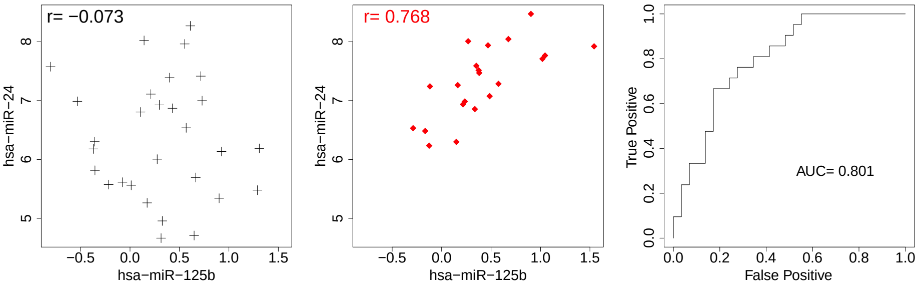

14:

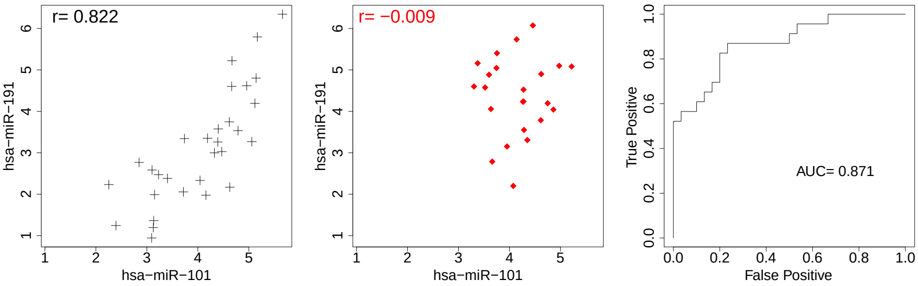

15:

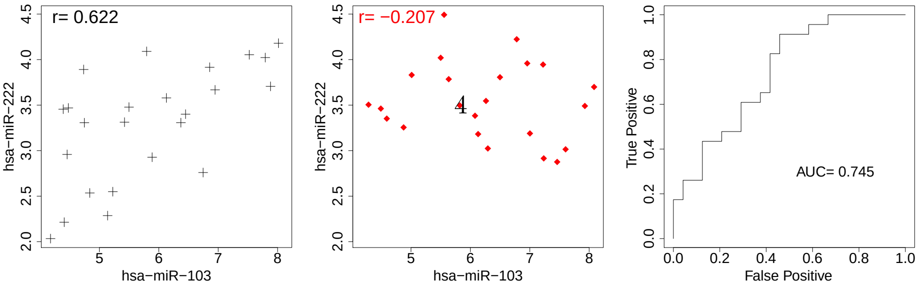

Figure 3: The scatterplots and ROC curves for the pairs of miRNAs selected by differential correlation analysis (part 3)

16:

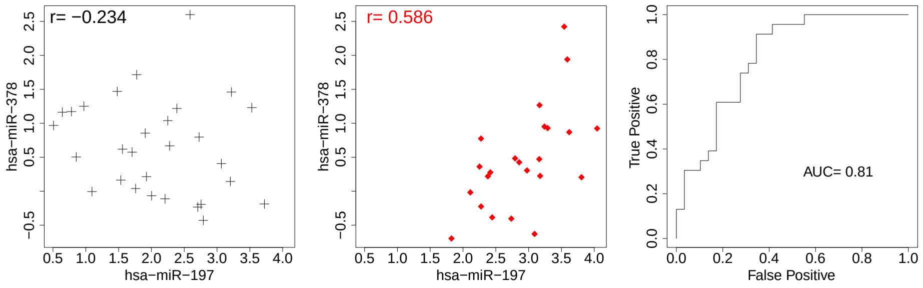

17:

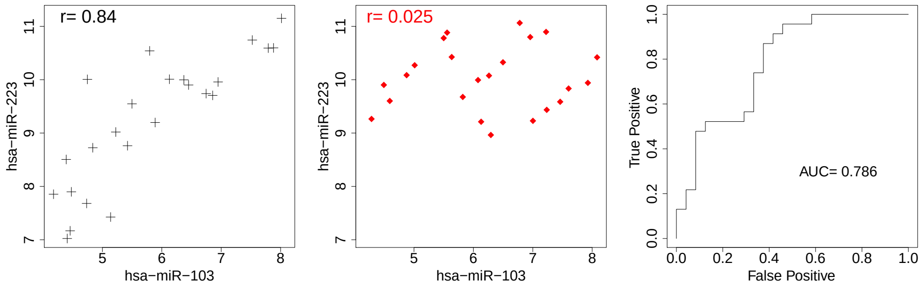

18:

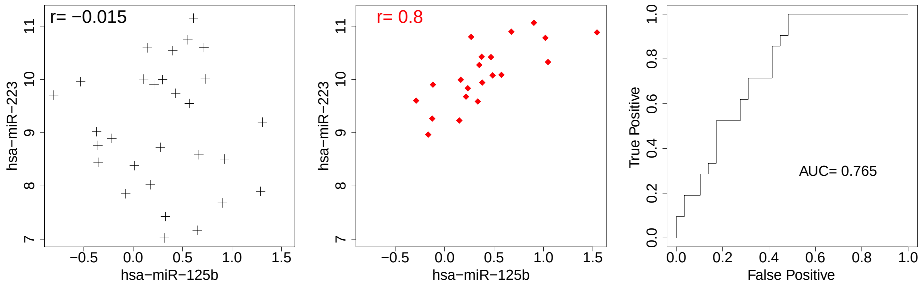

19:

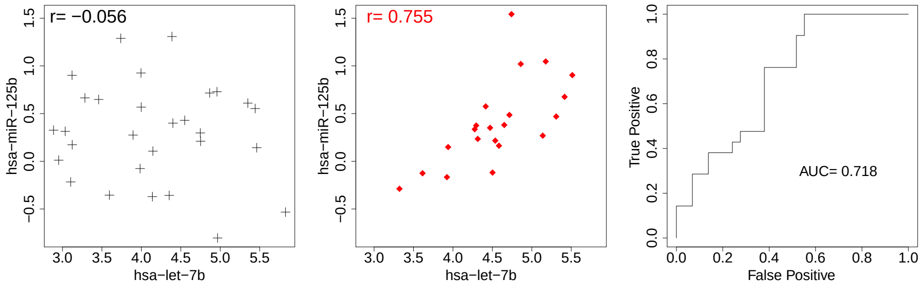

20:

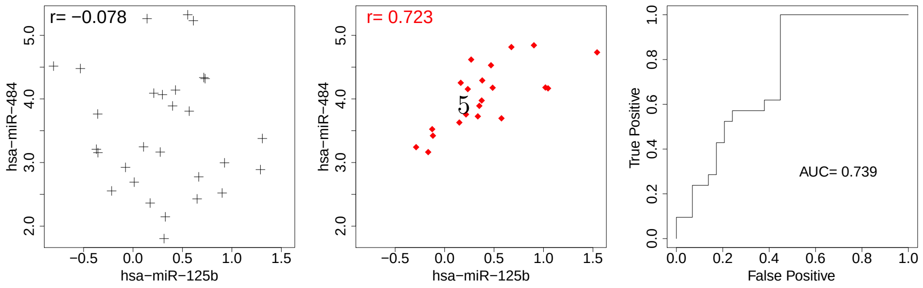

Figure 4: The scatterplots and ROC curves for the pairs of miRNAs selected by differential correlation analysis (part 4)
