# Supplementary material for: Plasma microRNA biomarker detection for mild cognitive impairment using differential correlation analysis
Source: Biomark Res. 2016 Dec 12;4:22. doi: 10.1186/s40364-016-0076-1 (PMC5151129; doi:10.1186/s40364-016-0076-1)
Supplement: Additional file 2 — Supplement B. Details of t-test and the results. The results include boxplots and ROC curves based on each of 22 miRNAs selected by t-test between Normal and MCI. (PDF 55.1 kb) [file 40364_2016_76_MOESM2_ESM.pdf]

Supplement B

Details of t-test and the results are shown. The results include boxplots and ROC curves of each of 22 miRNAs selected as significant biomarkers by t-test between Normal and MCI.

T-test: method

Traditional t-test searches MCI markers that are up- or down-regulated in MCI patients. In t-test, each miRNA has a p-value that is a measure of goodness of a biomarker. For example, a miRNA that has small p-value under .05 can be an up- or down-regulated in MCI patients. We can diagnose a candidate of patient according to the expression value of the miRNA. The goodness of miRNA as MCI markers is evaluated by AUC value based on the expression value of a miRNA, or equivalently on the estimated probabilities $\hat{p}_1, ..., \hat{p}_n$ that a sample is in MCI class, where $\hat{p}_1, ..., \hat{p}_n$ are estimated through logistic regression of $\log\{p/(1-p)\} = \beta_0 + \beta_1 X_1$, where β_0 and β_1 are regression coefficients and X_1 is the expression value of the miRNA. In order to evaluate the performance of multiple miRNAs simultaneously, the logistic regression below is applied:

$$\log \frac{p}{1-p} = \beta_0 + \beta_1 X_1 + \beta_2 X_2 + ... + \beta_k X_k \quad (1)$$

where $\beta_0, ..., \beta_k$ are regression coefficients and $X_1, ..., X_k$ are the expression value of k miRNAs. The performance of the k miRNAs as MCI markers can be evaluated by the AUC value in ROC analysis.

T-test: results

Traditional t-test was applied to the data set with 85 miRNAs for age-matched samples of 30 healthy controls and 23 MCI patients. The 22 miRNAs out of 85 were detected as biomarkers that significantly distinguish MCI from Normal (Table 1). ROC analysis was carried out to investigate the performance of each miRNA as a biomarker of MCI. The AUC (Area Under the Curve) value by each of the 22 miRNAs was 0.784 ± 0.017 ranged between 0.748 and 0.815. Figures 1 to 3 are boxplots and ROC curves based on each of 22 miRNAs that were selected as significant biomarkers by t-test between Normal and MCI. Also, AUC value for all four-miRNAs was calculated by using (1). The top four miRNAs (hsa-miR-151-3p, hsa-miR-30c, hsa-miR-23b and hsa-miR-144) attained high AUC value of 0.930 (Figure 4).

Table 1: Summary of the 22 miRNAs detected by t-test between Normal and MCI. The miRNAs are sorted by the p-value of the t-test. The mean AUC value is 0.784 ± 0.017.

Rank	miRNA	$\log_{10}$ p-value	AUC	Mean Expression Value ± Standard Error Normal	MCI
1	**hsa-miR-151-3p**	-5.26	0.828	2.05 ± 0.22	3.31 ± 0.12
2	**hsa-miR-126***	-4.51	0.800	4.27 ± 0.17	5.19 ± 0.13
3	**hsa-miR-23a**	-4.38	0.804	5.56 ± 0.16	6.47 ± 0.14
4	**hsa-miR-27b**	-4.25	0.794	3.31 ± 0.22	4.41 ± 0.14
5	**hsa-miR-146a**	-4.17	0.786	2.42 ± 0.22	3.51 ± 0.14
6	hsa-miR-30c	-4.12	0.794	2.18 ± 0.17	3.09 ± 0.14
7	hsa-miR-151-5p	-3.98	0.774	2.75 ± 0.22	3.87 ± 0.17
8	hsa-miR-23b	-3.95	0.784	3.11 ± 0.17	3.98 ± 0.14
9	hsa-miR-92a	-3.94	0.791	6.27 ± 0.10	6.82 ± 0.09
10	hsa-miR-24	-3.91	0.772	6.33 ± 0.19	7.26 ± 0.14
11	hsa-miR-144	-3.90	0.810	2.93 ± 0.20	1.87 ± 0.18
12	hsa-miR-15b	-3.86	0.783	4.25 ± 0.21	5.23 ± 0.14
13	hsa-let-7d*	-3.85	0.780	2.52 ± 0.15	3.30 ± 0.13
14	hsa-miR-197	-3.85	0.786	2.07 ± 0.17	2.92 ± 0.13
15	hsa-miR-30b	-3.83	0.790	2.60 ± 0.19	3.63 ± 0.18
16	hsa-miR-185	-3.68	0.766	3.33 ± 0.18	4.22 ± 0.14
17	hsa-miR-191	-3.65	0.778	3.11 ± 0.26	4.37 ± 0.21
18	hsa-miR-26b	-3.51	0.771	2.68 ± 0.19	3.64 ± 0.17
19	hsa-miR-223	-3.48	0.748	9.08 ± 0.22	10.03 ± 0.13
20	hsa-miR-26a	-3.47	0.764	4.64 ± 0.21	5.69 ± 0.19
21	hsa-miR-16	-3.32	0.771	6.50 ± 0.13	7.14 ± 0.12
22	hsa-let-7f	-3.30	0.780	0.96 ± 0.23	2.10 ± 0.23

Bold: top five miRNAs.

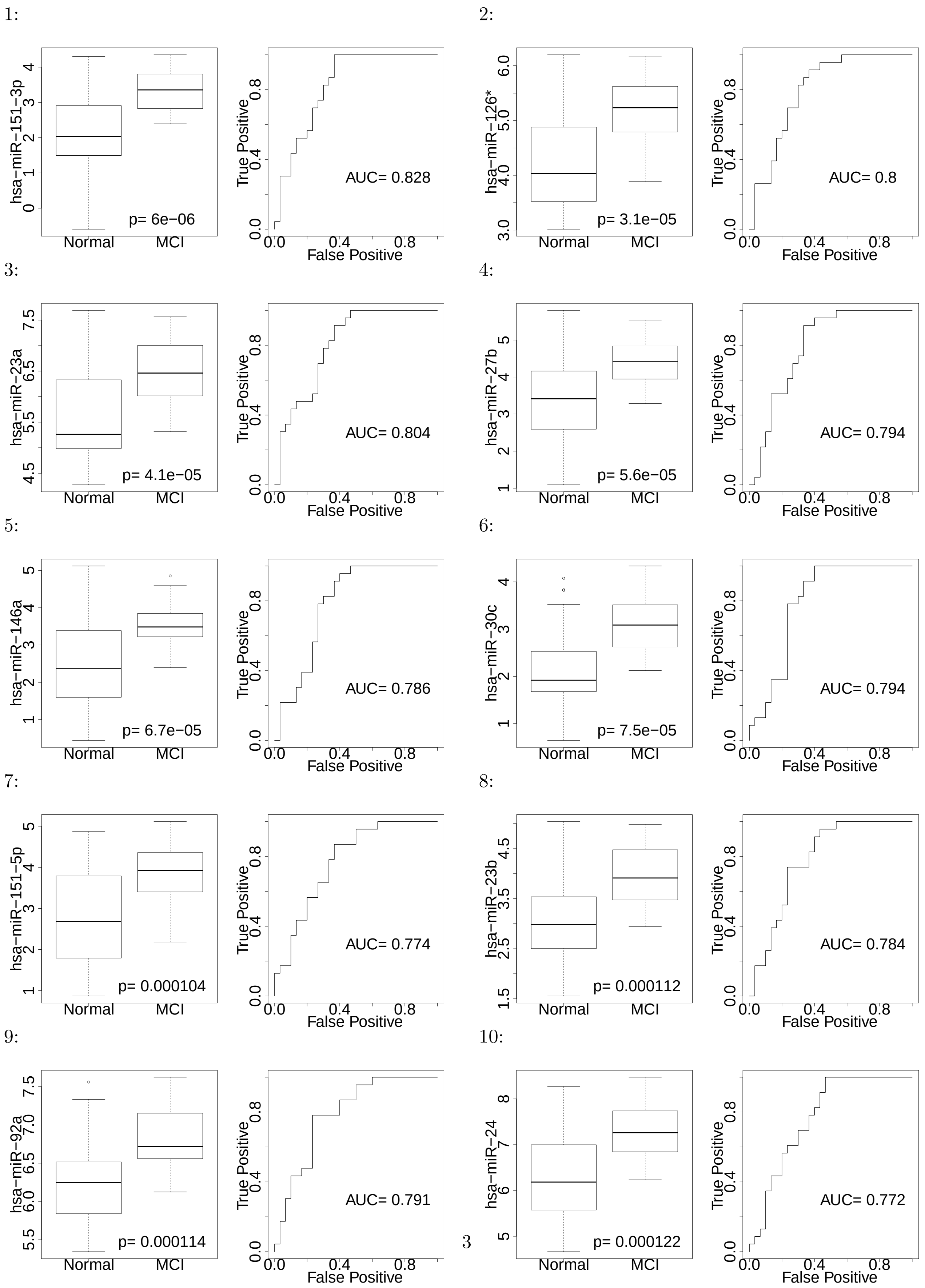

Figure 1: Boxplots and ROC curves for each of 22 miRNAs detected by t-test (part 1).

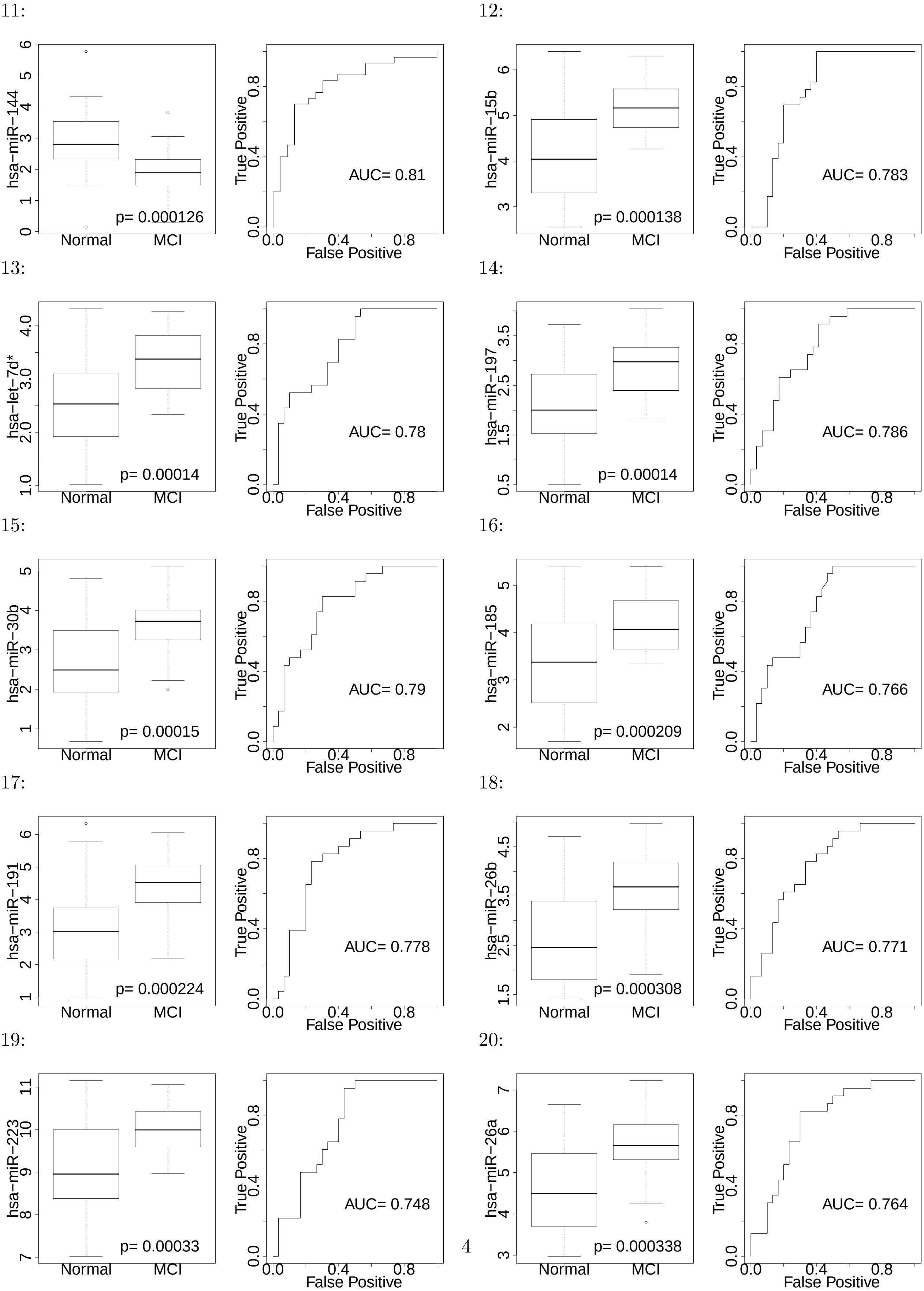

Figure 2: Boxplots and ROC curves for each of 22 miRNAs detected by t-test (part 2).

21: 22:

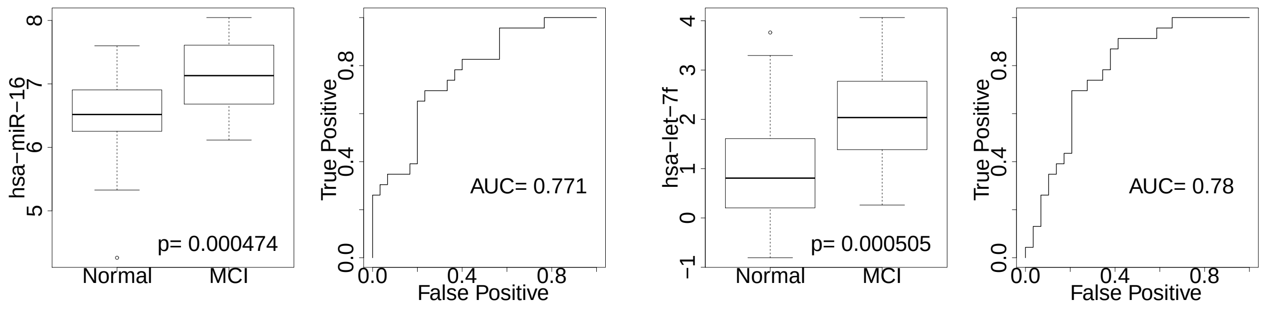

Figure 3: Boxplots and ROC curves for each of 22 miRNAs detected by t-test (part 3).

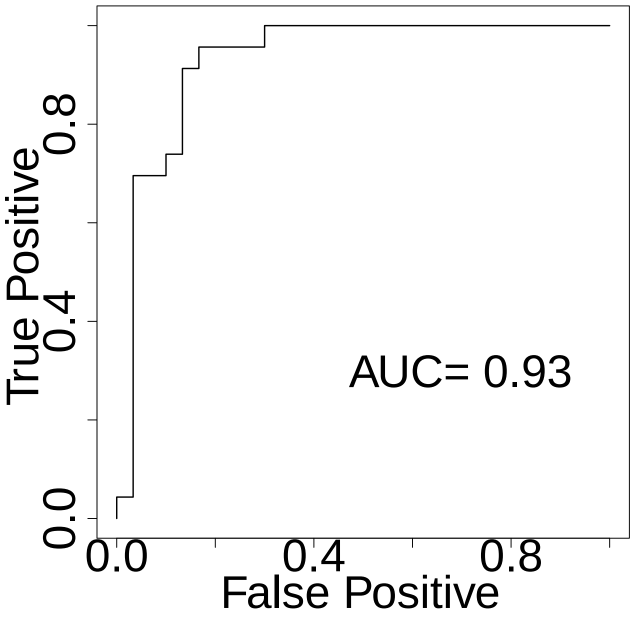

Figure 4: ROC curve based on the four miRNAs (hsa-miR-151-3p, hsa-miR-30c, hsa-miR-23b and hsa-miR-144) detected by t-test between Normal and MCI.
